# Autophagy-dependent changes in the sebaceous gland and epidermal lipid distribution localized by mass spectrometric imaging

**DOI:** 10.1016/j.xjidi.2026.100524

**Published:** 2026-08-11

**Authors:** Alexandra Stiegler, Samuele Zoratto, Christina Bauer, Bahar Golabi, Christopher Kremslehner, Sarah Jelleschitz, Ionela-Mariana Nagelreiter, Martina Marchetti-Deschmann, Florian Gruber

**Affiliations:** 1 Department of Dermatology, Medical University of Vienna, Vienna, Austria; 2 CDL SKINMAGINE, Vienna, Austria; 3 Institute of Chemical Technologies and Analytics, TU Wien, Vienna, Austria; 4 Zentrum für Hirnforschung, Medical University of Vienna, Vienna, Austria; 5 University of Veterinary Medicine, Vienna, Austria

**Keywords:** Autophagy, Lipid, Mass spectrometric imaging, Sebaceous gland

## Abstract

Sebum is a lipid mixture produced and delivered to the skin surface by appendages called sebaceous glands (SGs) in a holocrine secretion process. Sebum contributes to the skin barrier, lubricates hair and skin, provides antimicrobial protection, and has pheromone functions. During the terminal differentiation of SG cells (sebocytes), macroautophagy, an intracellular process for major metabolic reconfiguration, is activated. Deletion of autophagy affects the hair sebum composition in mice. This study aimed to investigate lipid composition and localization within SG and the epidermis in *Atg7* (autophagy related 7)-knockout (*atg7ΔKC*) and autophagy-competent mouse ears in situ using mass spectrometry imaging. Spatial lipidomics revealed lipid distribution with high spatial accuracy and identified lipid species with altered intensities in autophagy-deficient tissue. In SG regions of interest, signals putatively annotated as ceramide phosphate species (including ceramide phosphate 44:2;O_2_) showed higher signal intensities in *atg7ΔKC* mice. In contrast, selected sphingomyelin-associated signals showed lower intensities in the whole-tissue profile but not exclusively in SG regions of interest. It is also demonstrated that spatially resolved ion mass signatures are suitable for identifying the epidermis and SG in tissue ion images, a step toward “chemical analytical histology” of the skin.

## Introduction

The sebaceous gland (SG) forms the pilosebaceous unit together with the hair follicle. The SG is a unilobular or multilobular structure embedded in collagen-rich tissue, and its dominant cell type is sebocytes, which accumulate lipids in droplets during their maturation process (Mosca et al, 2025; Schneider, 2016). The final step of the maturation results in holocrine secretion of the sebum—a programmed cell death that releases the accumulated lipids. Holocrine secretion is a multistep, cell-specific process driven by DNase2 and linked to autophagy, which provides nutritional material (Fischer et al, 2017; Hossini et al, 2023; Zouboulis, 2017).

The released sebum is composed mainly of triacylglycerides, wax esters, and cholesterol esters but varies among species. Its primary function in mammals is the lubrication of skin and hair (Zouboulis et al, 2022). Further functions of sebum include providing insulation, antimicrobial protection, and pheromone production (Zouboulis et al, 2008). Spatial omics techniques, added to anatomical and functional aspects of the differentiating SGs, begins to allow a more holistic understanding of the microphysiological processes in this organ, and their impact is reviewed in Schmidt et al (2025). Especially high-resolution single-cell and spatial transcriptomics, as in Schmidt et al (2024) and Seiringer et al (2024), have identified relevant gene-regulatory programs and, using pseudotime ordering, allowed the arrangement of the few hundred sebocytes of a gland according to their maturation degree. These studies underlined that fatty-acid transport, lipid metabolism/de novo lipogenesis, the pentose phosphate pathway, hypoxia response, autophagy, and peroxisomal metabolism need to be activated as sebocytes progressively commit to sebum lipid synthesis, and in this study, we focused on the impact of macroautophagy on sebum lipid composition.

Macroautophagy, referred to as autophagy in the remaining parts of this paper, is an intracellular recycling mechanism that removes damaged macromolecules, protein aggregates, and organelles by sequestering them into autophagosomes, which later fuse with lysosomes, where the cargo is hydrolyzed. Autophagy is induced either under nutrient deprivation, where the breakdown products are supplied to the cell for resynthesis, or under metabolic stress, where it contributes to cellular quality control (Parzych and Klionsky, 2014; Yu et al, 2018). Autophagy that specifically promotes the degradation of intracellular lipid droplets is called lipophagy (Zhang et al, 2022).

A previous study in mice using a *K14* (keratin 14) promoter–driven Cre-LoxP system that deleted a floxed exon of the essential autophagy gene, *Atg7* (*atg7ΔKC*), showed increased amounts of lipids released to the skin surface as well as differences in lipid composition (Rossiter et al, 2018). Whereas cholesterol and free fatty acids were found to decrease, fatty acid methyl esters were elevated, as were wax esters of medium chain length contained in the sebum lipids. In addition, this study revealed an increased size of SG in the furry nape skin of the *atg7ΔKC* mice. Another study in mice of the same genotype focused on the preputial gland, a large gland situated on both sides of the penis. It is lined by sebocytes, which also secrete in a holocrine fashion, and the gland shows structural similarities to SG. The authors in that study applied matrix-assisted laser desorption/ionization (MALDI) mass spectrometry imaging (MSI) lipidomics and found a significant downregulation in phospholipid species within the preputial glands, specifically 1-palmitoyl-2-oleoyl-sn-glycero-3-phosphocholine (POPC) (Rossiter et al, 2022), which is a major component of lipid droplet membranes and can influence lipid droplet membrane curvature and size (Krahmer et al, 2011; Tauchi-Sato et al, 2002) and which was proposed to contribute to the observed anomaly of secreted droplets in the *atg7ΔKC*.

Because the spatial lipidome has not yet been resolved in the epidermis and SG of these mice, in this study, we investigated the effects of defective autophagy on lipid species in the SG and epidermis of ears from *atg7ΔKC* and autophagy-competent mice. We investigated differences in ear skin SG size to determine whether they compare with previous findings in furry nape skin (Rossiter et al, 2018). Furthermore, we utilized MALDI MSI to localize and map lipid species within the SG and the epidermis with high mass and spatial accuracy. Then, we investigated whether a tissue-specific molecular signature, defined by the combined spatial distribution and relative intensities of multiple lipid ions detected by MALDI MSI, could be used to differentiate the epidermis and SG from other tissue compartments. This could be important for the further development of chemical imaging technologies for histological applications, which seem feasible as the spatial resolution of MSI equipment increases, when localized ion marker combinations identifying specific cells or tissues could complement or even replace epitope recognition-based imagery.

## Results and Discussion

### SG size increased in young and old *atg7ΔKC* mouse ears

Previous studies reported that *K14*-driven deletion of *Atg7* in mice resulted in enlarged SG in back skin and early atrophy of the preputial glands (Rossiter et al, 2022, 2018).

**Figure 1:**
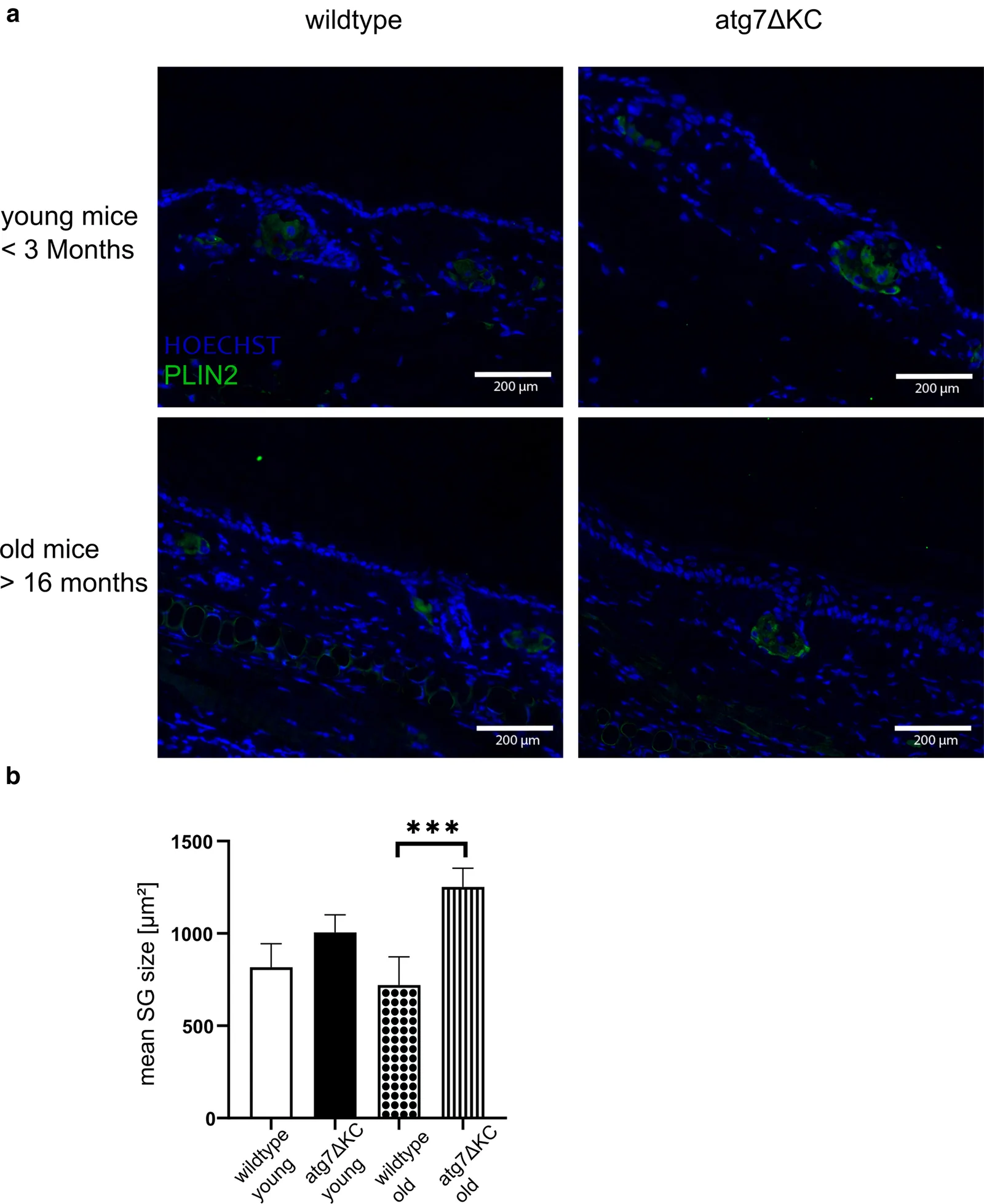
**Sebaceous gland size decreases in autophagy-deficient mouse ears.** (**a**) PLIN2-positive cells (green) indicate the location of the sebocytes within the sebaceous gland in ears harvested from young (aged <3 months) male wild-type and *Atg7ΔKC* and old (aged >16 months) male wild-type and *Atg7ΔKC* mice. Nuclei were stained with Hoechst-33258 dye. Bar = 200 μm. (**b**) Quantification of sebaceous gland size. Young *Atg7ΔKC* (animals, total sections, and total number of glands: 5, 28, and 752), young wild-type (5, 18, and 758), old *Atg7ΔKC* (3, 17, and 649), old wild-type (4, 17, and 510). One-way ANOVA with Sidak’s multiple comparison correction was used. Error bars indicate mean ± SD; ∗∗∗*P* < .005.

In this study, we investigated SG in both old (aged >16 months) and young (aged <3 months) male *atg7ΔKC* and wild-type mouse ears, which we later also investigated with MALDI MSI imaging. We visualized SGs by staining paraffin-embedded tissue sections with perilipin 2, which stains the lipid droplets stored in sebocytes (Dahlhoff et al, 2013), using anti-PLIN2 (Figure 1a), and used Fiji (ImageJ) to measure SG size.

We identified an increase in the mean SG size in young *atg7ΔKC* mice (5 animals, 28 sections, 752 glands) compared with that in young wild-type mice (5 animals, 18 sections, 758 glands), which did not reach statistical significance, whereas we identified a significant increase in SG size in old *atg7ΔKC* mice (3 animals, 17 sections, 649 glands) compared with that in old wild-type mice (4 animals, 17 sections, 510 glands) (1-way ANOVA, *P* = .0004) (Figure 1b).

Our results corroborate the previously reported increase in SG size in the nape skin of *atg7ΔKC* mice (Rossiter et al, 2018). This indicates that the increase in SG size detected in *atg7ΔKC* mice is present in multiple tissues. Although the changes did not reach significance, the observed size increase in young *atg7ΔKC* mice compared with that in autophagy-competent mice indicates that the knockout results in phenotypic differences starting from a young age and that these differences increase with age. Our findings add to the characterization of *atg7ΔKC* mice because our young mice were harvested at the age <3 months, whereas in the work of Rossiter et al (2018), the SGs were investigated in mice aged 9–14 months.

### MALDI MSI mapping of epidermal and SG lipids in *atg7ΔKC* mouse ears

**Figure 2:**
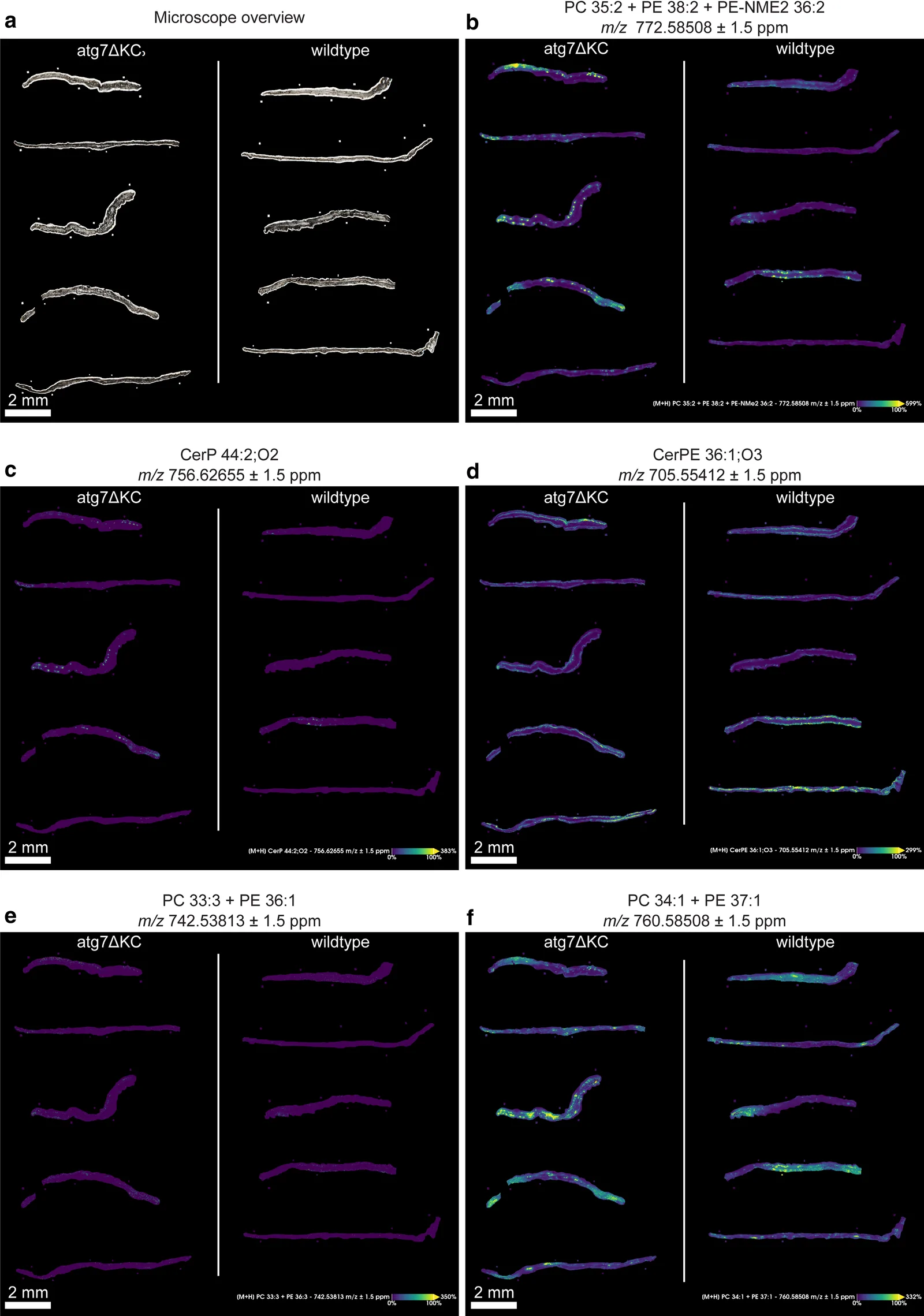
**Overview of mass spectrometric imaging localization of selected lipid-associated signals in all measured samples in *atg7ΔKC* and wild-type mice.** (**a**) Microscopic overview of samples taken before MALDI MSI was measured. MALDI-FTICR-MSI ion images showing the distribution of (**b**) m/z 772.58508 (putatively annotated as PC 35:2), (**c**) m/z 756.62655 (putatively annotated as CerP 44:2;O2), (**d**) m/z 705.55412 (putatively annotated as CerPE 36:1;O3), (**e**) m/z 742.53813 (putatively annotated as PC 33:3), and (**f**) m/z 760.58508 (putatively annotated as PC 34:1) in the positive ion mode. Color bars show the relative matrix-peak–normalized signal intensity for each ion, scaled from 0% (black) to 100% (yellow). The percentage printed next to each color bar denotes the highest detected intensity relative to the upper display limit. Bars = 2 mm. CerP, ceramide phosphate; CerPE, ceramide phosphoethanolamine; MALDI, matrix-assisted laser desorption/ionization; MALDI-FTICR-MSI, matrix-assisted laser desorption/ionization Fourier transform ion cyclotron resonance mass spectrometry imaging; MSI, mass spectrometry imaging; PC, phosphatidylcholine; PE, phosphatidylethanolamine; PE-NME2, dimethylphosphatidylethanolamine.

The *Atg7* knockout in mice has been reported to cause SG hyperplasia, and a changed lipid profile was assessed using classical lipidomic methods, including thin-layer chromatography and MALDI MS on epidermal surface lipids extracted from hair (Rossiter et al, 2018). A subsequent study in the preputial glands of *Atg7*-deficient mice found that holocrine lipid secretion was disrupted. Using MALDI MSI, the investigators localized aberrant phospholipid composition in areas containing differentiating sebocytes within preputial glands, which could explain the aberrant formation of lipid droplets in those glands (Rossiter et al, 2022). We utilized MALDI MSI to investigate the SG lipidome of aged (>16 months) *atg7ΔKC* mouse ears with enlarged SG, compared with that of wild-type controls in situ. An overview of selected lipid-associated signals in *atg7ΔKC* and wild-type mice is shown in Figure 2. At the same time, we tried to establish a workflow to characterize murine skin using the spatial distribution of lipid ions detectable by MSI. This is relevant for future developments in histology that could omit staining using antibodies or dyes and would be based on chemical imaging.

**Figure 3:**
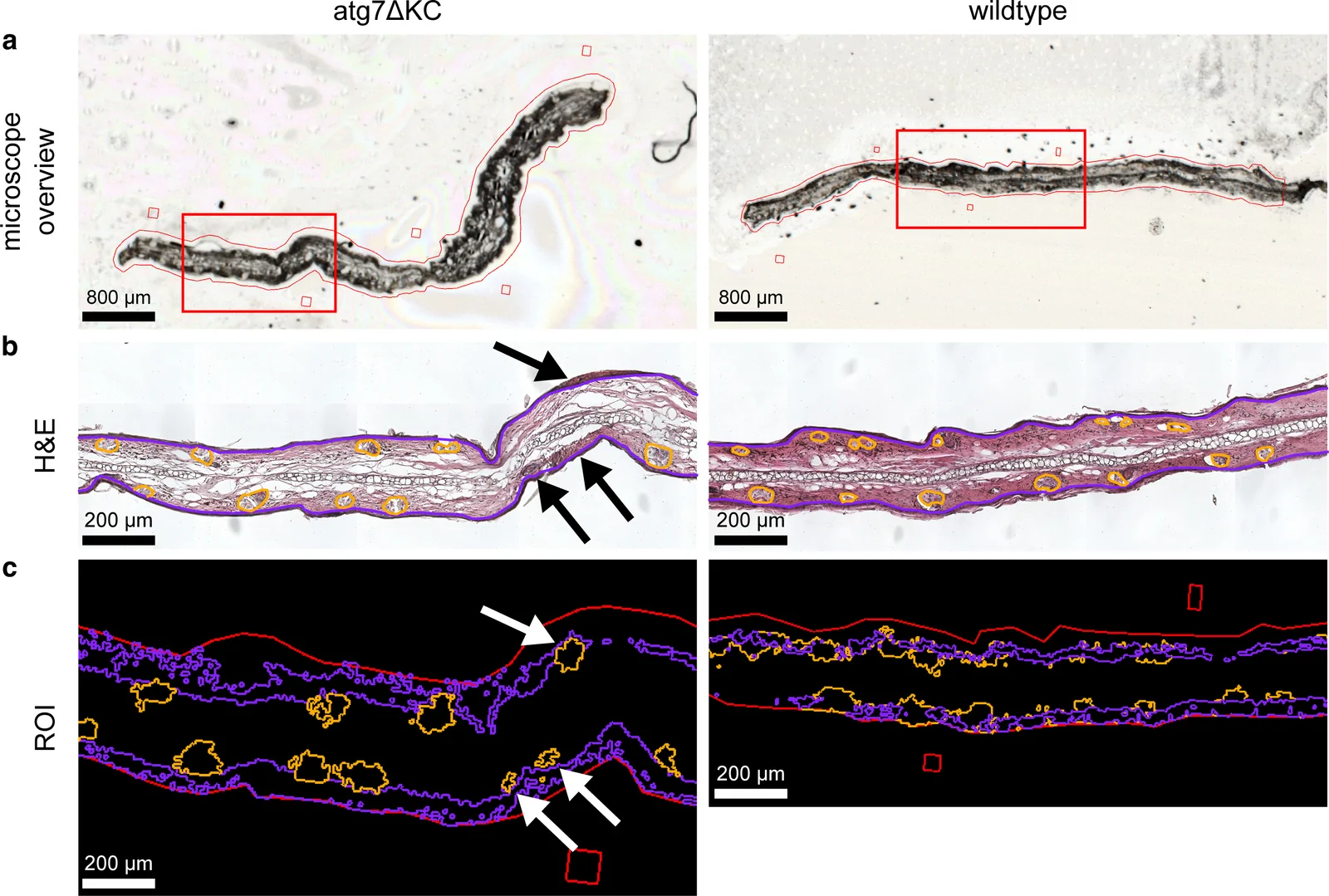
**The software correctly identifies epidermis and sebaceous gland regions comparable to those of a trained researcher.** (**a**) Microscopic overview of samples taken before MALDI MSI was measured. The red box indicates the sections selected for zooming into the samples. (**b**) Zoomed in H&E staining of cryosections of *Atg7ΔKC* and wild-type mouse ears. The purple line indicates the basal layer of the epidermis. The yellow circles indicate identified sebaceous glands. The black arrow indicates an area of the section where a sebaceous gland is located but not identified by the investigator because of tissue folding. (**c**) Software identification of epidermis and sebaceous glands in ROIs. The purple line indicates the identified epidermis. The yellow circled areas identify the sebaceous glands. The white arrow indicates a sebaceous gland identified by the software, which was missed by the investigator owing to tissue folding. Bars (**a**) = 800 μm and (**b** and **c**) = 200 μm. MALDI, matrix-assisted laser desorption/ionization; MSI, mass spectrometry imaging; ROI, region of interest.

**Figure 4:**
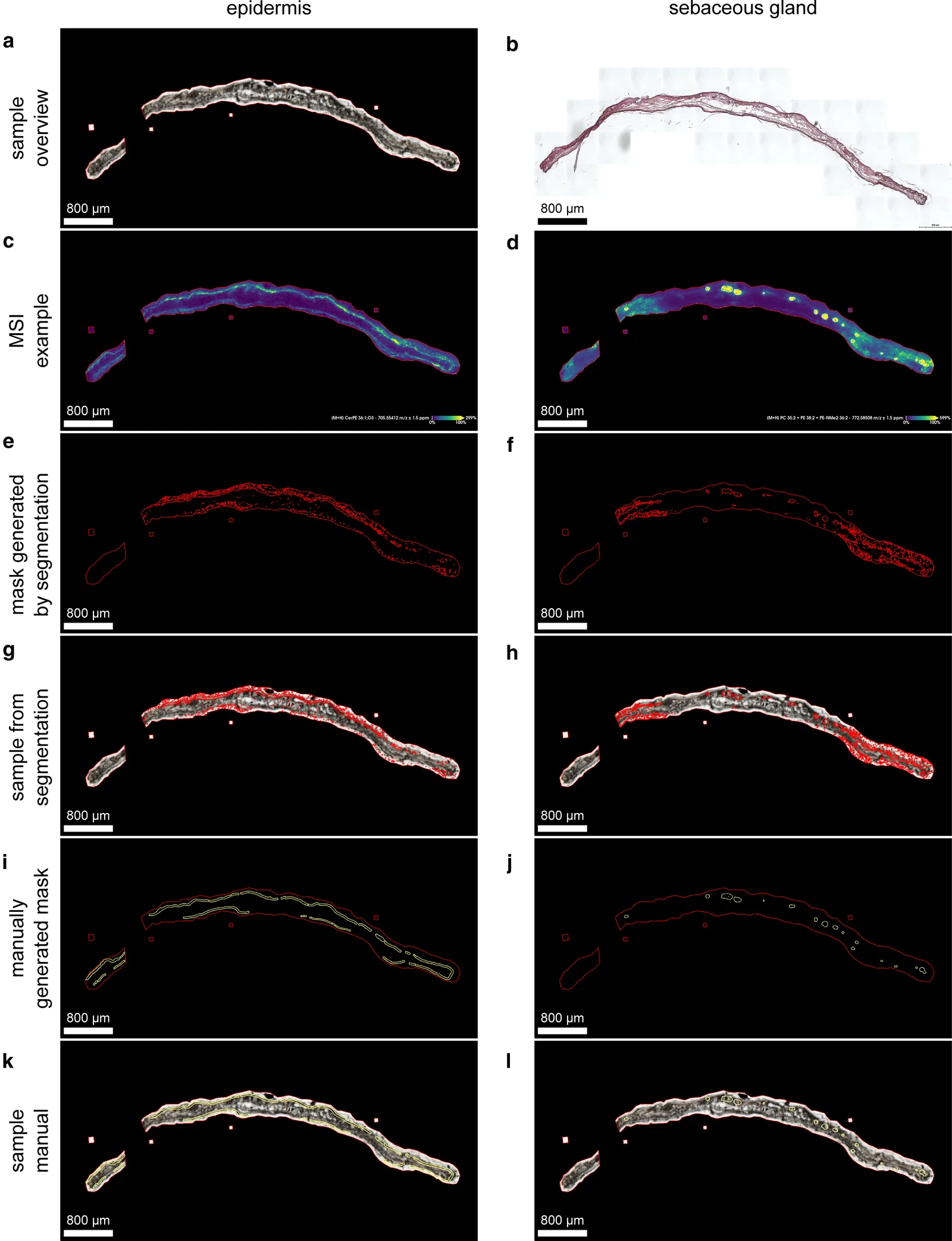
**Epidermis and sebaceous gland ROIs were corrected manually when the segmentation algorithm failed to correctly identify them.** (**a**) Sample overview taken before MALDI MSI was measured. (**b**) H&E staining after MALDI MSI measurement. (**c, d**) MSI results sample lipids used for manual correction. (**e, f**) ROI mask generated by segmentation algorithm. (**g**, **h**) Overlay of microscope image and mask generated by segmentation. (**i, j**) Mask generated manually by trained investigator. (**k**, **l**) Overlay of microscopy image and manually generated ROI mask. Bar = 800 μm. MALDI, matrix-assisted laser desorption/ionization; MSI, mass spectrometry imaging; ROI, region of interest.

We used SCiLS (2026b Pro, Bruker) to automate the identification of the epidermis and SG area of our samples. For each sample, segmentation was calculated from a 3-dimensional Uniform Manifold Approximation and Projection of features matched against an internal library derived from the LipidMAPS database. We inspected the resulting segmentation and used only the areas matching the epidermis or SG to generate the region of interest (ROI). Visible in the alignment with the microscope overview (Figure 3a) and the H&E sections examined by trained investigators (Figure 3b), SCiLS produced region outlines that agreed with the expected tissue structures (Figure 3c). In cases where the ion intensity was low or the segmentation included adjacent tissue, the regions were manually corrected. Figure 4 shows the tissue masks for epidermis (left panels) or SGs (right panels) generated either by segmentation (Figure 4e–h) or by manual input (Figure 4i–l). Notably, SCiLS identified correct regions that had been overlooked by the investigators owing to folding of the specimen (Figure 3b and c, arrows).

**Figure 5:**
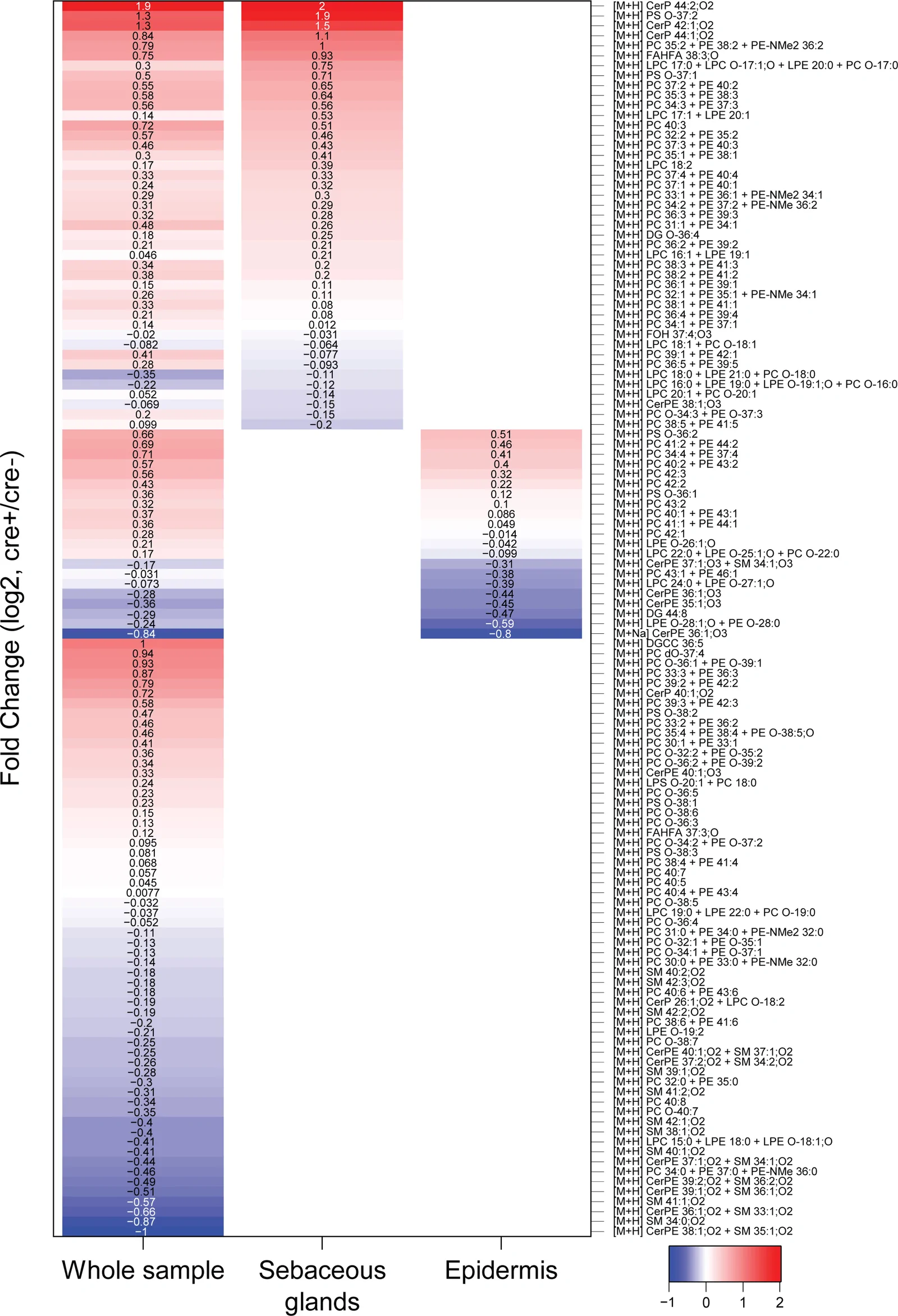
**FC calculation of measured differences in signal intensity showed lipid species–specific deregulation in *Atg7ΔKC* mouse ear epidermis and sebaceous glands.** The positive FC (red) indicates higher lipid expression in *Atg7ΔKC* mice than in wild-type mice, and the negative FC (blue) indicates lower lipid expression in *Atg7ΔKC* mice than in wild-type mice. White indicates an FC near zero, and black pattern indicates that the signal was too low for the specific lipid within the epidermis. EPI denotes epidermis, cre+ denotes *Atg7ΔKC*, and cre− denotes wild type. FC, fold change; SG, sebaceous gland.

**Figure 6:**
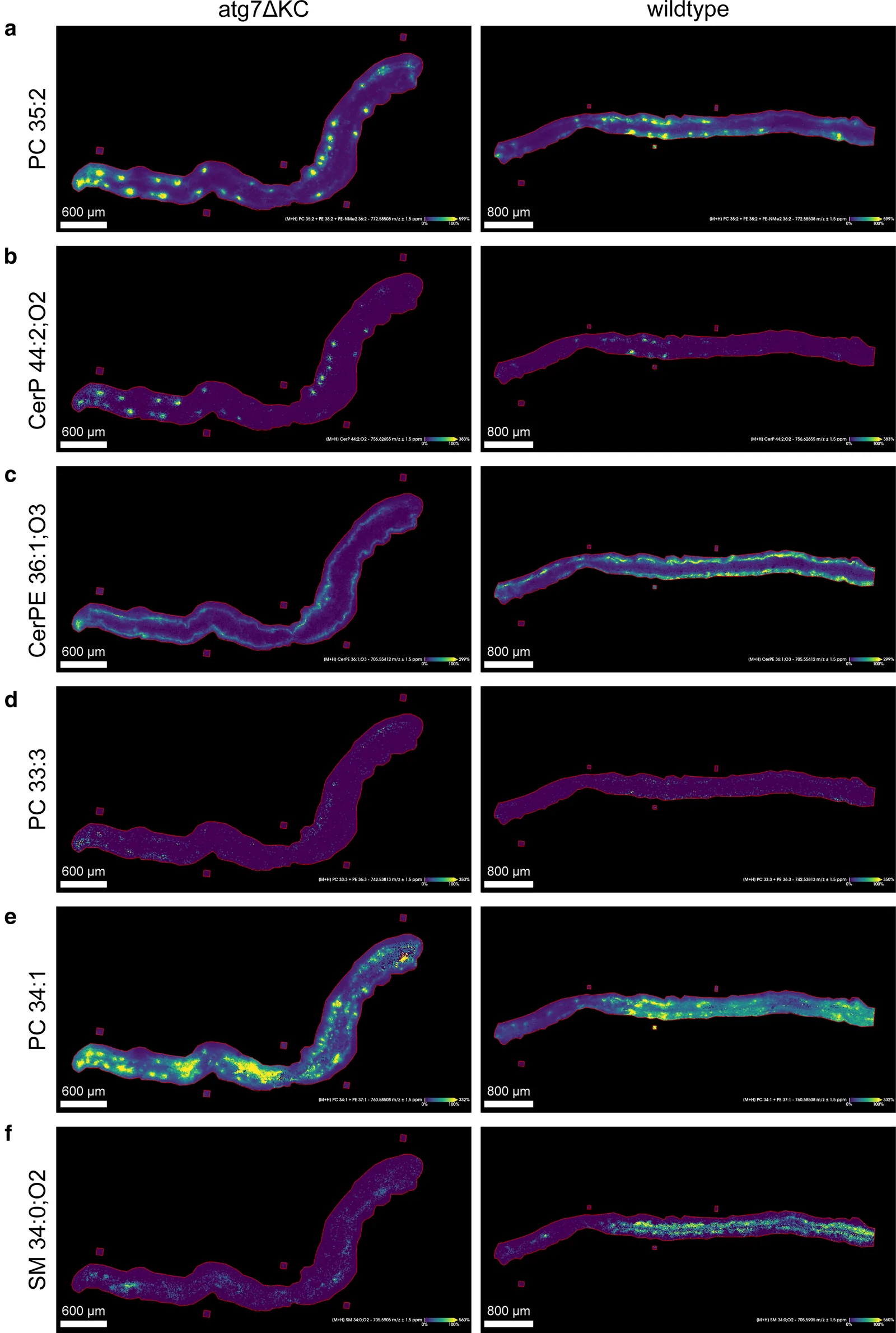
**Mass spectrometric imaging locates selected lipid-associated signals in epidermis and sebaceous glands.** MALDI-FTICR-MSI ion images showing the distribution of (**a**) m/z 772.58508 (putatively annotated as PC 35:2), (**b**) m/z 756.62655 (putatively annotated as CerP 44:2;O2), (**c**) m/z 705.55412 (putatively annotated as CerPE 36:1;O3), (**d**) m/z 742.53813 (putatively annotated as PC 33:3), (**e**) m/z 760.58508 (putatively annotated as PC 34:1), and (**f**) m/z 705.5905 (putatively annotated as SM 34:0;O2) in the positive ion mode. Color bars show the relative matrix-peak–normalized signal intensity for each ion, scaled from 0% (black) to 100% (yellow). The percentage printed next to each color bar denotes the highest detected intensity relative to the upper display limit. Bar (left panels) = 600 μm and (right panels) = 800 μm. CerP, ceramide phosphate; CerPE, ceramide phosphoethanolamine; MALDI-FTICR-MSI, matrix-assisted laser desorption/ionization Fourier transform ion cyclotron resonance mass spectrometry imaging; PC, phosphatidylcholine; SM, sphingomyelin.

We used the identified ROIs to calculate the log_2_ fold change (FC) of lipid intensities in *atg7ΔKC* mice relative to that in the control mice (3 donors per group, 5 samples) (Figure 5 and Supplementary Table S1). Lipid-associated m/z values were interpreted as compartment specific only when they were reliably detected in the corresponding ROI-specific analysis. Accordingly, blank areas in Figure 5 indicate features that were present in the whole sample but were not retained for the respective SG or epidermal ROI and were not interpreted as ROI-specific changes. In SG ROIs, several signals showed higher mean intensities in the *atg7ΔKC* mice. Among these, the signal at m/z 772.58508, putatively annotated by accurate mass as phosphatidylcholine (PC) 35:2, and the signal at m/z 756.62655, putatively annotated as ceramide phosphate (CerP) 44:2;O_2_, were predominantly localized in the SG (Figure 6a and b). Both signals showed higher mean intensities in SG ROIs of *atg7ΔKC* mice than in wild-type animals, with CerP 44:2;O_2_ showing the largest SG-associated change (log_2_ FC = 2.03).

**Figure 7:**
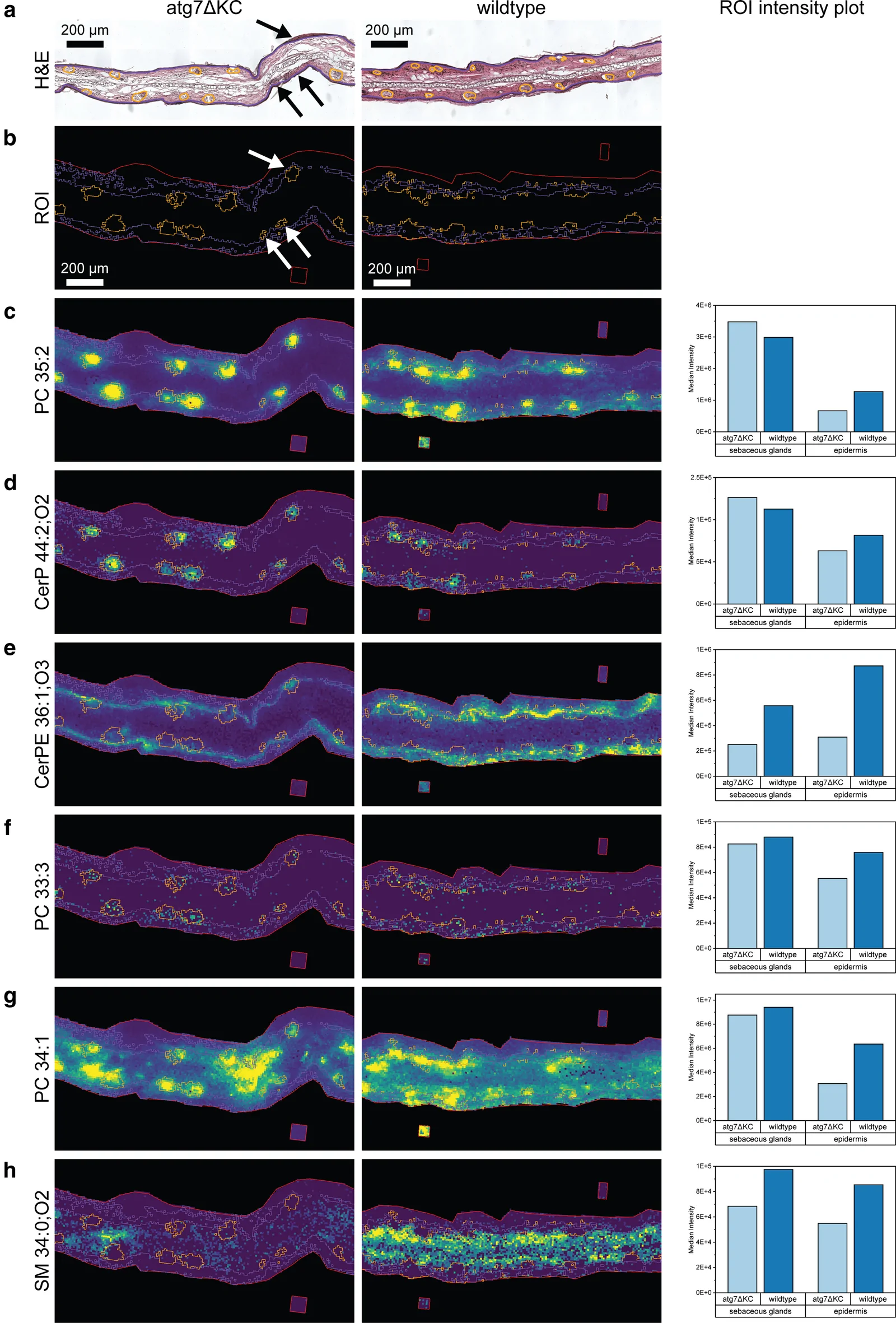
**Zoomed MSI visualization and ROI-based intensity comparison of selected lipid-associated signals in epidermis and sebaceous glands.** (**a**) H&E images and (**b**) corresponding ROI outlines show the zoomed tissue areas from 1 representative *atg7ΔKC* and 1 representative wild-type sample. MALDI-FTICR-MSI ion images show the distribution of (**c**) m/z 772.58508 (putatively annotated as PC 35:2), (**d**) m/z 756.62655 (putatively annotated as CerP 44:2;O2), (**e**) m/z 705.55412 (putatively annotated as CerPE 36:1;O3), (**f**) m/z 742.53813 (putatively annotated as PC 33:3), (**g**) m/z 760.58508 (putatively annotated as PC 34:1), and (**h**) m/z 705.5905 (putatively annotated as SM 34:0;O2) in positive ion mode. Bar plots show the median ion intensity within the displayed epidermis and sebaceous gland ROIs and are intended as visual ROI comparisons, not as statistical analyses across biological replicates. Bars = 200 μm. The panels **c**–**h** are enlarged outtakes from Figure 6 aligned to the consecutive sections shown in Figure 3. CerP, ceramide phosphate; CerPE, ceramide phosphoethanolamine; MALDI-FTICR-MSI, matrix-assisted laser desorption/ionization Fourier transform ion cyclotron resonance mass spectrometry imaging; MSI, mass spectrometry imaging; PC, phosphatidylcholine; ROI, region of interest; SM, sphingomyelin.

Within the epidermis, the signal at m/z 705.55412, putatively annotated as ceramide phosphoethanolamine 36:1;O_3_, was retained in the epidermis-specific ROI and showed lower mean intensity in *atg7ΔKC* (log_2_ FC = −0.44) (Figure 6c). A sodiated ceramide phosphoethanolamine 36:1;O_3_ candidate showed a strong epidermal decrease (log_2_ FC = −0.80), and its colocalization is consistent with the hydrogenated species. The signal at m/z 742.53813, putatively annotated as PC 33:3, was shown as a spatial example (Figure 6d) but was not retained in either the SG or epidermal ROIs. Furthermore, we detected the signal at m/z 760.58508, putatively annotated as PC 34:1, with comparable intensities across genotypes in the SG ROI (log_2_ FC = 0.01) and only a modest change in the whole-tissue analysis (log_2_ FC = 0.14) (Figure 6e). This annotation includes POPC as one possible molecular species. The signal at m/z 705.5905, putatively annotated as sphingomyelin (SM) 34:0;O2, is the most strongly reduced one with a unique annotation in the *atg7ΔKC* tissues (Figure 6f). The zoomed-in images of these species (Figure 7) show how they align with the ROI marks, and in the case of SM 34:0;O2, it is observed that most of the signal stems from the dermal tissue outside the ROIs. For several of the annotations, phosphoethanolamine or diacylglycerophosphoethanolamine variants could not be completely excluded on the basis of accurate mass. Because we acquired the data in positive ion mode, these alternatives are unlikely; yet, they are listed in Figure 5 for completeness.

Using SCiLS to identify regions in *atg7ΔKC* mouse ears enabled us to highlight the differences in lipid localization between the epidermis and SG. A similar approach has been applied in human skin to map lipid markers within distinct zones of the pilosebaceous unit (Xie et al, 2022). In this study, the automated identification of the SG closely matched the regions annotated on the H&E-stained section; we therefore considered the computationally defined SG and epidermal regions to be equivalent to those defined by manual inspection.

Sebum composition is species specific. In mice, sebum consists mainly of wax esters with a smaller fraction of triacylglycerides (Vietri Rudan and Watt, 2022) and also contains ceramides (Franco et al, 2018). In our study, lipid-associated signals found in the SG and epidermis were considered compartment specific only when reliably detected in the corresponding ROI. Thus, whole-tissue signals or visually present low-intensity signals were not interpreted as SG or epidermal changes unless they were retained in the respective ROI-specific column of Figure 5. That most SG lipids are traceable in the epidermis is consistent with the biology of the pilosebaceous unit, because sebocytes accumulate lipids and release them through holocrine secretion through the hair canal and onto the skin surface (Schneider, 2016).

Within each region, we examined whether *atg7* deficiency was associated with intensity trends among lipid-associated signals. Because SGs in *atg7ΔKC* mouse ear were significantly enlarged, we used the mean pixel intensity within each ROI to avoid bias introduced by differences in gland size. No individual lipid species reached statistical significance; however, in SG ROIs, signals putatively annotated as CerP candidates, such as CerP 44:2;O_2_, CerP 42:1;O_2_, and CerP 44:1;O_2_, showed a consistent trend toward higher mean intensities in *atg7ΔKC* mouse ears. In contrast, SM-associated signals were not retained in the SG ROI analysis. SM candidates, such as SM 34:0;O_2_, SM 42:1;O_2_, SM 41:1;O_2_, and SM 40:2;O_2_, showed lower mean intensities in the whole-tissue profile and should therefore be interpreted as global rather than SG-specific trends. Rossiter et al (2022) previously reported that ceramides and SM were unchanged in ATG7-deficient mouse preputial glands, whereas all phospholipid classes were significantly downregulated, and diacylglycerols were significantly upregulated. In this study, we detected the same phospholipid classes, but we did not observe the same uniform downregulation in *atg7ΔKC* mice tissues (Figure 5).

We detected the signal at m/z 760.58508, putatively annotated as PC 34:1, at high intensity in both genotypes. PC 34:1 includes POPC as one possible molecular species, which is of interest because POPC is a major component of lipid droplet membranes and limits droplet coalescence, thereby influencing droplet size (Krahmer et al, 2011; Tauchi-Sato et al, 2002). In contrast to the decrease in POPC reported in *atg7ΔKC* mouse preputial glands (Rossiter et al, 2022), we did not observe a meaningful difference in the PC 34:1 candidate signal between wild-type and *atg7ΔKC* mouse ears (Figure 6e) in the SG ROI or whole-tissue analysis. Because the assignment was based on accurate mass, the exact molecular species contributing to this signal remains tentative. Both studies used the same MALDI MSI acquisition methodology, so the differences in findings may reflect differences between the 2 tissues analyzed and/or differences in the age of the animals. In our study, mice were killed after more than the age of 16 months, whereas in the study by Rossiter et al (2022), the preputial glands were obtained from mice aged 10–14 months.

Our MALDI MSI results were obtained in positive ion mode to enable measurements at 10 μm spatial resolution. Given the small tissue area and the complete rastering of the available sample, additional analysis in negative ion mode on the same section was not possible. Therefore, we were unable to corroborate or localize the findings previously found in *atg7ΔKC* mouse epidermis using high-performance liquid chromatography-tandem mass spectrometry analysis in negative ion mode, in which the investigators reported a significant reduction of triacylglycerides in *Atg7*-deficient mice (Yang et al, 2022). Future MALDI MSI studies in this model will benefit additionally from combining positive and negative ion modes to improve lipid class coverage. Among the limitations was variability in overall lipid intensities observed between samples of the same genotype. Although normalization to a matrix peak was applied, and acquisition order was randomized, the extended measurement time required for high-resolution Fourier transform ion cyclotron resonance MSI necessitated analysis across multiple days. Day-to-day variations in experimental conditions as well as differences in tissue quality and section thickness may therefore have contributed to the observed intersample variability.

The findings of this study confirm that defective autophagy was associated with increased SG size in old mice. Although no individual lipid species showed statistically significant differences between *atg7ΔKC* and wild-type mice, the high-resolution MSI data allowed us to map lipid distributions within the epidermis and the SG with high spatial accuracy. The consistent trends observed across samples suggest that loss of autophagy may affect the local lipid environment, even if the changes remain modest at the level of individual species. In SG ROIs, signals putatively annotated as CerP candidates showed higher mean intensities in *atg7ΔKC* mice, whereas selected SM candidates showed lower mean intensities in the whole-tissue profile but were not retained in the SG ROI analysis. These findings may indicate changes in sphingolipid-related signals and may contribute to altered SG homeostasis in *atg7ΔKC* mice (Miner et al, 2025). From a technical perspective, 10 μm spatial lipidomics with MALDI MSI enables the localization of lipid-associated signals within defined small skin compartments. This approach complements (immuno) histological analysis by providing molecular information directly within tissue structures and supports the spatial investigation of metabolic processes in situ.

## Materials and Methods

### Mice

The *Atg7*^*F/F*^ *GFP-LC3* mice, which were used as the control group, and the *atg7*^*F/F*^ *K14-Cre GFP-LC3* mice (termed “a*tg7ΔKC*” or “mutant” test mice), in which *Atg7* was inactivated, and therefore, autophagy was disrupted, have been described previously (Rossiter et al, 2022, 2013; Song et al, 2017; Sukseree et al, 2020, 2012; Yang et al, 2022).

Mice were maintained and killed by cervical dislocation according to the animal welfare guidelines of the Medical University of Vienna (Vienna, Austria), as approved by the Ethics Review Committee for Animal Experimentation of the Medical University of Vienna and the Federal Ministry of Science, Research and Economy (Austria). Genomic DNA preparation and genotyping to identify K14-Cre, LoxP, and GFP-LC3–bearing mice were carried out as described previously (Rossiter et al, 2018, 2013). The immunofluorescent staining was performed on young male mice (aged <3 months) and old male mice (aged >16 months). For MALDI MSI, only old male mice were used.

### Histology

The antibody guinea pig anti-adipophilin (PLIN2, BP5012, 1:100, Acris, OriGene, Rockville, MD) was used to stain SG. The corresponding secondary fluorescently labeled antibody, Alexa Fluor 488 (A11073, Invitrogen, Waltham, MA), was used at a 1:500 dilution. The samples were counterstained with 2 μg/ml Hoechst-33258 (H1398, Thermo Fisher Scientific, Waltham, MA)

Both ears were collected from killed mice and either directly embedded in optimal cutting temperature medium (Scigen, Singapore, Singapore) or were fixed, dehydrated, and embedded in paraffin and then cut at a thickness of 5 μm and stained as previously described (Rossiter et al, 2022).

Ears embedded in optimal cutting temperature were cut into sections 6–7, 6–7 μm thick onto ITO slides (Shimadzu, Kyoto, Japan). After MALDI MSI, the samples were stained with H&E according to standard procedures.

Images for all sections were captured at ×20 magnification on a TissueFAXs (AX10, TissueGnostics, Vienna, Austria) equipped with 1-color camera (HXG40c, Baumer Group, Frauenfeld, Switzerland) and 1 fluorescence camera (Orca Flash 4.0, Hamamatsu, Shizuoka, Japan).

The anti-PLIN2–stained fluorescence images were analyzed in Fiji (ImageJ, National Institutes of Health, Bethesda, MD) by marking the SG with the polygon selection tool and measuring the area in μm^2^. The measurements were compiled into an Excel file, and statistical analysis and graphical representation were done in GraphPad Prism 8.4.3 (GraphPad Software, Boston, MA).

To calculate statistical significance, a 1-way ANOVA with the Šidák multiple-comparison correction was used. Statistical significance was indicated by asterisks, with ∗∗∗ indicating *P* < .005.

### MALDI imaging mass spectrometry

Fresh frozen samples embedded in optimal cutting temperature were sectioned at 6–7 μm at the Medical University of Vienna and mounted on ITO-coated glass slides. Optimal cutting temperature was not removed because it did not interfere with the selected mass range. Sections were hand delivered on ice and stored at −70° C until analysis. Before matrix application, the slides were thawed for 45 minutes in a desiccator at room temperature to avoid moisture deposition. Optical images were acquired with a PrimeHisto XE slide scanner (Pacific Image Electronics).

Matrix deposition was performed by sublimation using a home-built device, as described in Holzlechner et al (2017). A total of 15 mg of 1,5-DAN was dissolved in 7 ml of acetone, corresponding to a coating of approximately 0.2 mg/cm^2^ on the tissue. Sublimation was carried out at 140° C for 5 minutes under a vacuum of 5.0 × 10^-2^ mbar with a cold finger set to 10° C. After coating, slides were kept in a room-temperature desiccator for at least 24 hours before measurement.

MALDI MSI was performed on a Bruker scimaX MRMS 7T instrument (Bruker Daltonics, Billerica, MA) operated in positive ion mode. Data were acquired in 2-omega mode with a 4 M data word size over a mass range of m/z 150–1200. The SmartBeam II laser (355 nm) was operated at 100 Hz with 10 shots per pixel and a 9.4% fluence. A lateral resolution of 10 μm was used without oversampling. External linear calibration was performed using red phosphorus spotted on the same slide.

All datasets were processed in SCiLS Lab 2026b Pro (SCiLS GmbH, Bremen, Germany). Matrix-related normalization was carried out using the reference peak at m/z 317.1761. Default hotspot removal was applied, and no denoising or interpolation was performed. Tentative lipid assignments were obtained by accurate-mass matching against an internal list derived from the LipidMAPS database. These annotations should be considered putative and, where applicable, refer to lipid class and sum composition. Isobaric species, isomeric species, different acyl-chain arrangements, and alternative adducts cannot be excluded without tandem mass spectrometry validation. In total, 5 slides comprising 10 samples were analyzed.

## Ethics Statement

Animal procedures were approved by the Ethics Review Committee for Animal Experimentation of the Medical University of Vienna (Vienna, Austria) (approval numbers BMWF-66.009/0124-II/10b/2010 and subsequent). No experiments on live animals were performed.

## Data Availability Statement

The authors affirm that all data necessary for confirming the conclusions of the article are present within the article, figures, and tables.

## ORCIDs

Alexandra Stiegler: http://orcid.org/0009-0005-9034-0264

Samuele Zoratto: http://orcid.org/0000-0002-1491-3404

Christina Bauer: http://orcid.org/0009-0001-1867-1596

Bahar Golabi: http://orcid.org/0000-0002-0974-8280

Christopher Kremslehner: http://orcid.org/0000-0001-9746-196X

Sarah Jelleschitz: http://orcid.org/0009-0003-6036-8093

Ionela-Mariana Nagelreiter: http://orcid.org/0000-0003-4684-7947

Martina Marchetti-Deschmann: http://orcid.org/0000-0002-8060-7851

Florian Gruber: http://orcid.org/0000-0003-1094-5641

## Conflict of Interest

The authors state no conflict of interest.
